# Better outcomes after minimally invasive surgeries compared to the standard invasive medial parapatellar approach for total knee arthroplasty: a meta-analysis

**DOI:** 10.1007/s00167-020-06306-9

**Published:** 2020-10-06

**Authors:** Filippo Migliorini, Jörg Eschweiler, Alice Baroncini, Markus Tingart, Nicola Maffulli

**Affiliations:** 1 Department of Orthopaedics, University Clinic Aachen, RWTH Aachen University Clinic, Pauwelsstraße 30, 52074 Aachen, Germany; 2 Department of Medicine, Surgery and Dentistry, University of Salerno, Via S. Allende, 84081 Baronissi, SA Italy; 3 Barts and The London School of Medicine and Dentistry, Centre for Sports and Exercise Medicine, Mile End Hospital, Queen Mary University of London, 275 Bancroft Road, London, E1 4DG England; 4 School of Pharmacy and Bioengineering, Keele University Faculty of Medicine, Thornburrow Drive, Stoke on Trent, England

**Keywords:** Total knee arthroplasty, Minimally invasive surgery, Standard invasive surgery

## Abstract

**Purpose:**

Minimally invasive surgery (MIS) for total knee arthroplasty (TKA) is often marketed as being able to speed up healing times over standard invasive surgery (SIS) through the medial parapatellar approach. The advantages of these minimally invasive approaches, however, are not yet definitively established. A meta-analysis of studies comparing peri-operative and post-operative differences and long-term complications of MIS versus SIS for TKA was conducted.

**Methods:**

This meta-analysis was conducted following the PRISMA guidelines. The Pubmed, Google Scholar, Scopus, and Embase databases were accessed in September 2020. All clinical trials comparing minimally-invasive versus standard approaches for TKA were considered. Only studies reporting quantitative data under the outcomes of interest were included. Methodological quality assessment was performed using the PEDro appraisal score.

**Results:**

This meta-analysis covers a total of 38 studies (3296 procedures), with a mean 21.3 ± 24.3 months of follow-up. The MIS group had shorter hospitalization times, lower values of total estimated blood loss, quicker times of straight-leg raise, greater values for range of motion, higher scores on the Knee Society Clinical Rating System (KSS) and its related Function Subscale (KSFS). Pain scores, anterior knee pain and revision rate were similar between MIS and SIS. SIS allowed a quicker surgical duration.

**Conclusion:**

The present meta-analysis encourages the use of minimally invasive techniques for total knee arthroplasty. However, MIS TKA is technically demanding and requires a long learning curve.

**Level of evidence:**

III, meta-analysis of clinical trials.

## Introduction

The most common exposure for total knee arthroplasty (TKA) is the medial parapatellar arthrotomy (MPP) using standard incision surgery (SIS) [22]. Despite the satisfying surgical outcomes, this approach has been criticized as it produces extensive damage to the knee extensor muscle mechanism, and it may negatively affect the patellar blood supply [45]. Thus, a less invasive MPP which allowed to spare the quadriceps (quadriceps-sparing approach = QS) [30] and the limited- or mini-medial parapatellar approach (MMPP) [50]. The midvastus and subvastus [59] approaches became respectively the mini-midvastus (MMV) [15], and the mini-subvastus (MSV) [17]. MIS for TKA uses a surgical incision shorter than 14 cm, thus offering an attractive alternative for both surgeons and patients. Despite the large number of published studies comparing the MIS and standard approaches, there is still lack of consensus concerning the best approach for TKA. Under these premises, a meta-analysis comparing MIS versus the traditional MPP SIS approach for TKA was conducted, investigating outcomes and long-term complications between the two approaches. The goal of the present study is to update current evidence and offer new insights concerning the surgical exposure to the orthopaedic surgeons.

It was hypothesised that MIS for TKA may achieve superior surgical outcomes than the MPP SIS approach.

## Material and methods

### Search strategy

This meta-analysis was performed according to the Preferred Reporting Items for Systematic Review and Meta-Analysis (PRISMA guidelines) [39]. The search parameters were defined as follows:

(P) Population: patients requiring TKA;

(I) Intervention: SIS TKA though the MPP approach;

(C) Comparison: MIS TKA;

(O) Outcomes: peri-operative data, functional scores, complications.

The search was performed in September 2020. The databases accessed were Pubmed, Google Scholar, Scopus, and Embase, without any limitation on time of publication. The following keywords were used: total knee arthroplasty, total knee replacement, prosthesis, combined with minimally-invasive, medial parapatellar, mini-medial parapatellar, minivastus, subvastus, quadriceps-sparing, and further combined with anterior knee pain, revision, range of motion, scores, blood loss, surgical duration, outcomes. Two independent authors (**; **) performed the database search. If title and related abstract matched the topic, the full-text article was accessed. The bibliographies for each article of interest were screened by hand. Disagreements between the authors were debated and solved.

### Eligibility criteria

Two independent authors (**; **) screened articles for inclusion. All clinical trials comparing minimally-invasive approaches for TKA to the standard approach were considered for inclusion. According to the authors’ capabilities, articles in English, French, Spanish, Italian, and German were included. Only clinical trials with evidence levels I to III were considered according to the Oxford Centre of Evidenced-Based Medicine [23]. Every type of TKA (cruciate or bi-cruciate retaining, posterior stabilized) was considered eligible. No distinction was made between different MIS approaches. Studies taking advantage of a navigation system were also included. Case series, reviews and meta-analyses, editorials and expert opinions were excluded. Biomechanical, in vitro, animal and cadaveric studies were also excluded. Only studies reporting quantitative data under the outcomes of interest were included.

### Outcomes of interest

Two independent authors (**, **) screened the included studies and extracted the following generalities: year, type of study, number of knees, duration of follow-up (in months), surgical approach, percentage of osteoarthritic and female patients, mean age and body mass index (BMI) (kg/m^2^). For each approach, the following peri-operative endpoints were collected: duration of surgery and hospitalization, total estimated blood loss (intra-operative and post-operative). Functional scores included range of motion (ROM), knee flexion, time of straight-leg raise (SLR), the Knee Society Clinical Rating System (KSS) and its related Function Subscale (KSFS) [42] and the visual analogic scale for pain (VAS). Procedure-related complications, anterior knee pain and need for revision were also retrieved.

### Methodological quality assessment

For methodological quality assessment, the PEDro scale was applied. This scale is a validated system for evaluating the quality of clinical trials [38]. Two authors (**; **) who already had extensive experience with this score independently evaluated each article. The PEDro scale evaluates studies based on the criteria: clearly eligibility criteria, allocation, baseline comparability, blinding, follow-up, analyses, point estimates and variability. A final mean value > 6 is considered to indicate good methodological quality.

### Statistical analysis

The statistical analysis was performed by the main author (**). For the assessment of baseline comparability, the IBM SPSS Software was used. The unpaired *t*-test was performed, with values of *P* > 0.5 considered satisfactory. Statistical analyses were performed using Review Manager Software 5.3 (the Nordic Cochrane Collaboration, Copenhagen). For continuous variables, the inverse variance method with mean difference (MD) effect measure was adopted, while for binary data, the Mantel–Haenszel method with Odds Ratio (OR). The confidence interval was set to 95% in all comparisons. A fixed effect was set as default for every comparison. Heterogeneity was assessed through the Chi-square (*χ*^2^) and Higgins Tests (*I*^2^). If *χ*^2^ > 0.5, the *I*^2^ test was evaluated. *I*^2^ test values of 25, 50 and 75% detected respectively low, moderate and high levels of heterogeneity. If high heterogeneity was detected, a random effect model was used. The forest and funnel plot were performed to establish a visual representation of the effect measure and risk of publication bias, respectively.

## Results

### Literature search

**Fig. 1:**
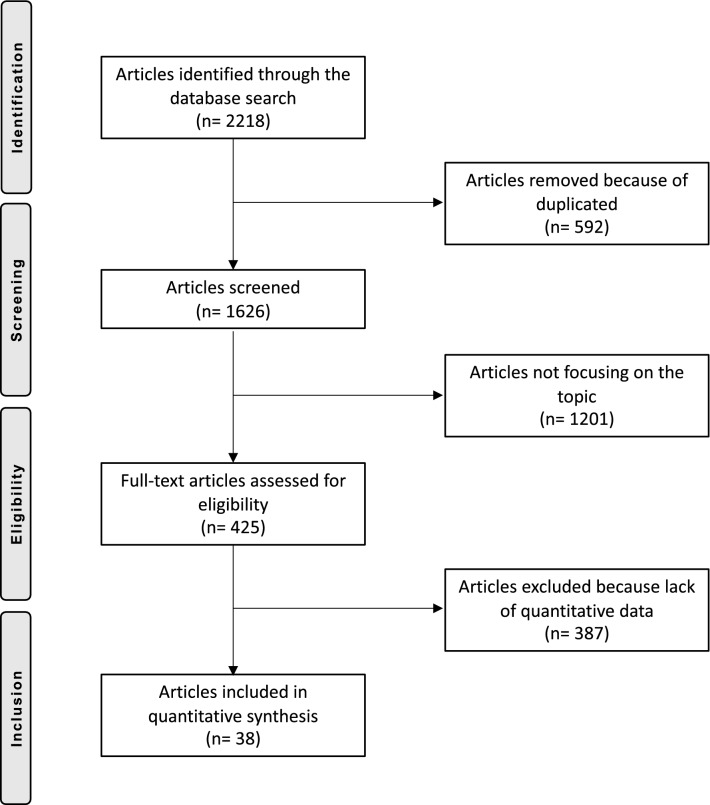
Flow-chart of the literature search

The initial literature search resulted in 2218 articles, of which 592 were duplicates. 1201 did not match the eligibility criteria and a further 371 did not report quantitative data under the outcomes of interest. Another 16 articles were excluded because of uncertain results or untrustworthy data origin. In the end, this left 38 articles for inclusion: 22 RCTs and 16 n-RCTs. Figure 1 shows the flow-chart of the literature search.

### Methodological quality assessment

The mean PEDro score for included studies showed high quality at 7.3/10. The main limitation reflected in this score results from the impossibility of blinding the surgeons. This score evidenced that in 27% (10/38) of studies the follow-up was too short and 24% (9/38) did not provide any randomization of the samples. The PEDro score assigned to each study is shown in Table 1.

**Table 1:** PEDro methodological assessment score

Author	1	2	3	4	5	6	7	8	9	10	11	Total
Aslam et al. 2017 [4]	Y	Y	Y	Y	Y	N	Y	Y	Y	Y	Y	10
Avci et al. 2013 [5]	Y	N	N	Y	N	N	N	Y	Y	Y	Y	6
Boerger et al. 2005 [6]	Y	N	N	Y	N	N	N	N	Y	Y	Y	5
Bridgman et al. 2009 [7]	Y	Y	Y	Y	N	N	Y	Y	Y	Y	Y	9
Chalidis et al. 2010 [8]	Y	Y	Y	Y	N	N	N	Y	Y	Y	Y	8
Chiang et al. 2012 [9]	Y	Y	Y	Y	Y	N	Y	Y	Y	Y	Y	10
Cho et al. 2014 [10]	Y	Y	Y	Y	N	N	N	Y	Y	Y	Y	8
Dabboussi et al. 2012 [13]	Y	N	N	Y	N	N	N	N	Y	Y	Y	5
Feczko et al. 2016 [18]	Y	Y	Y	Y	N	N	N	N	Y	Y	Y	7
Han et al. 2008 [20]	Y	Y	Y	Y	N	N	Y	Y	Y	Y	Y	9
Hernandez-Vaquero et al. 2010 [22]	Y	Y	Y	Y	N	N	N	N	Y	Y	Y	7
Huang et al. 2015 [24]	Y	N	N	Y	N	N	N	Y	Y	Y	Y	6
Jung et al. 2009 [25]	Y	N	N	Y	N	N	N	Y	Y	Y	Y	6
Juosponis et al. 2009 [26]	Y	Y	Y	Y	N	N	Y	N	Y	Y	Y	8
Karachalios et al. 2008 [27]	Y	Y	Y	Y	N	N	N	Y	Y	Y	Y	8
Karpman et al. 2009 [28]	Y	Y	Y	Y	N	N	Y	N	Y	Y	Y	8
Kim et al. 2011 [29]	Y	Y	Y	Y	Y	N	Y	Y	Y	Y	Y	10
King et al. 2007 [31]	Y	N	N	Y	N	N	N	N	Y	Y	Y	5
Laskin et al. 2004 [33]	Y	N	N	Y	N	N	N	N	Y	Y	Y	5
Li et al. 2017 [34]	Y	Y	Y	Y	N	N	N	Y	Y	Y	Y	8
Liebensteiner et al. 2012 [35]	Y	N	N	Y	N	N	N	N	Y	Y	Y	5
Mehta et al. 2017 [40]	Y	Y	Y	Y	N	N	N	N	Y	Y	Y	7
Rahman et al. 2015 [43]	Y	N	N	Y	N	N	N	N	Y	Y	Y	5
Schroer et al. 2008 [46]	Y	N	N	Y	N	N	N	Y	Y	Y	Y	6
Seon et al. 2007 [47]	Y	N	N	Y	N	N	N	Y	Y	Y	Y	6
Stevens-Lapsley et al. 2012 [48], 2013 [14]	Y	Y	Y	Y	N	N	Y	N	Y	Y	Y	8
Tasker et al. 2014 [49]	Y	Y	Y	Y	N	N	Y	Y	Y	Y	Y	8
Tenholder et al. 2005 [50]	Y	N	N	Y	N	N	N	Y	Y	Y	Y	6
Thienpont et al. 2013 [51]	Y	Y	Y	Y	N	N	N	Y	Y	Y	Y	8
Tsuji et al. 2010 [53]	Y	N	N	Y	N	N	N	N	Y	Y	Y	5
Unnanuntana et al. 2012 [54]	Y	N	N	Y	N	N	N	Y	Y	Y	Y	6
Unwin et al. 2017 [55]	Y	Y	Y	Y	N	N	N	Y	Y	Y	Y	8
Varela-Egocheaga et al. 2009 [56]	Y	Y	Y	Y	N	N	N	Y	Y	Y	Y	8
Watanabe et al. 2009 [57]	Y	N	N	Y	N	N	N	Y	Y	Y	Y	6
Wegrzyn et al. 2013 [58]	Y	Y	Y	Y	Y	N	Y	N	Y	Y	Y	10
Wülker et al. 2010 [60]	Y	Y	Y	Y	N	N	N	Y	Y	Y	Y	8
Zhu et al. 2015 [62]	Y	N	N	Y	N	N	N	Y	Y	Y	Y	6

1. Eligibility criteria; 2. Random allocation; 3. Concealed allocation; 4. Baseline comparability; 5. Blind subject; 6. Blind clinician; 7. Blind assessor; 8. Adequate follow-up; 9. Intention-to-treat analysis; 10. Between-group analysis; 11. Point estimates and variability

### Risk of publication bias

**Fig. 2:**
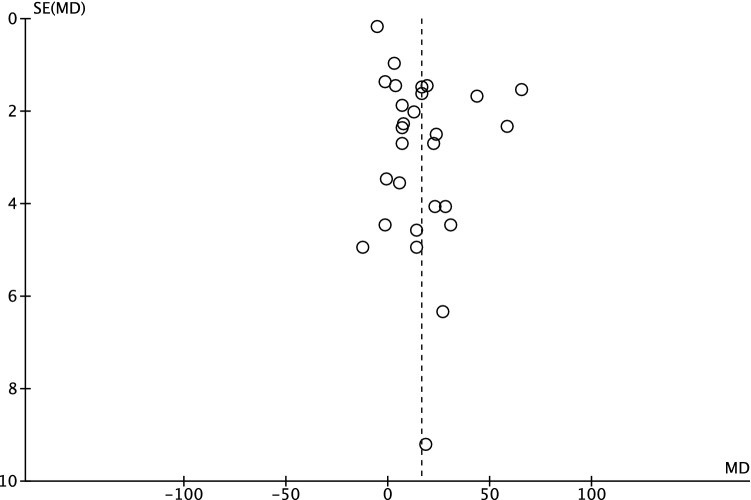
Funnel plot of the most reported outcome, surgical duration

To evaluate the risk of publication bias, the funnel plot of the most reported outcome (surgical duration) was performed. The plot (Fig. 2) shows a moderate symmetrical distribution of the referral points. There is adequate distribution with respect to the no-effect line. Consequently, this meta-analysis shows a moderate risk of publication bias for.

### Patient demographic

In the present study, data from 3281 patients were collected. The mean follow-up time was 21.3 ± 24.3 months. 1697 patients had undergone TKA using a MIS approach, and 1584 patients using the SIS approach. There was baseline comparability between age, BMI, gender and diagnosis (*P* > 0.5). Study generalities and patient demographic are shown in Table 2, while Table 3 shows in detail the baseline characteristic of the two cohorts.

**Table 2:** Study generalities and demographic baseline of the enrolled patients

Author, year	Type of Study	Knees (*n*)	Follow-up (months)	Type of approach	Knees (*n*)	Osteoarthritis (%)	Female (%)	Mean age (years)	BMI (kg/m^2^)
Aslam et al. 2017 [4]	RCT	84	12	MMV	42	100	30	68.8	30.6
				MPP	42	100	57	68.6	30.1
Avci et al. 2013 [5]	RCT	39	23.5	MMV	19	100	79	64.5	32.0
Boerger et al. 2005 [6]	n-RCT	120	3	MSV	60	100	77	69.0	28.0
				MPP	60	100	75	68.0	29.0
Bridgman et al. 2009 [7]	RCT	224	13	MSV	113		48	70.1	
				MPP	111		49	70.9	
Chalidis et al. 2010 [8]	RCT	100	24	MMV	50	100	92	70.1	34.6
				MPP	50	100	88	71.2	34.2
Chiang et al. 2012 [9]	RCT	75	24	QS	38	100	90	69.7	28.6
				MPP	37	100	90	69.8	29.6
Cho et al. 2014 [10]	RCT	66	12	MMV	33	100	96	65.5	29.1
				MPP	33	100	94	67.0	28.0
Dabboussi et al. 2012 [13]	n-RCT	80	3	MMV	40	100			
				MPP	40	100			
Feczko et al. 2016 [18]	RCT	69	6	MMV	36	95	64	65.1	28.3
				MPP	33	100	67	64.9	28.6
Han et al.2008 [20]	RCT	30	24	MMPP	15	100		66.0	26.9
				MPP	15	100		64.0	26.4
Hernandez-Vaquero et al. 2010 [22]	RCT	62	6	MMV	26	100	81	70.8	32.1
				MPP	36	100	80	70.5	30.8
Huang et al. 2015 [24]	n-RCT	96	60	MMPP	35	100	86	69.2	27.0
				QS	31	100	94	69.3	26.9
				MPP	30	100	93	71.2	26.7
Jung et al.2009 [25]	n-RCT	40	58.4	MSV	21				
				MPP	19				
Juosponis et al. 2009 [26]	RCT	70	3	MMV	35	100	86	72.0	28.0
				MPP	35	100	86	71.4	29.1
Karachalios et al. 2008 [27]	RCT	100	23	MMV	50	92	62	71.1	32.0
				MPP	50	92	70	70.8	31.5
Karpman et al. 2009 [28]	RCT	59	6	MMV	20	100	65	74.0	30.0
				QS	20	100	60	73.0	28.0
				MPP	19	100	53	73.0	29.0
Kim et al. 2011 [29]	RCT	50	12	MMV	23	100		67.0	27.1
				MPP	22	100		68.0	28.4
King et al. 2007 [31]	n-RCT	150	1.5	QS	100	95	52	67.0	30.0
				MPP	50	90	66	28.0	32.0
Laskin et al. 2004 [33]	n-RCT	58	3	MMV	26			70.0	30.0
				MPP	26			68.0	29.0
Li et al. 2017 [34]	RCT	50	12	MSV	25	100	64	69.9	25.8
				MPP	25	100	64	68.1	25.5
Liebensteiner et al. 2012 [35]	n-RCT	38	2	MMV	19		58	66.7	30.2
				MPP	19		53	67.6	31.5
Mehta et al. 2017 [40]	RCT	55	6	MSV/MMV	26		73	59.8	
				MPP	29		73	61.4	
Rahman et al. 2015 [43]	n-RCT	120	3	MMPP	60	100	75	59.8	
				MPP	60	100	77	62.0	
Schroer et al. 2008 [46]	n-RCT	300	24	QS	150		62	71.0	31.0
				MPP	150		61	70.0	32.0
Seon et al. 2007 [47]	n-RCT	84	12	MMV	41	100	80	64.2	
				MPP	43	100	77	64.2	
Stevens-Lapsley et al. 2012 [48], 2013 [14]	RCT	41	3	MMPP	22	100	54	64.6	30.5
				MPP	19		45	64.0	31.3
Tasker et al. 2014 [49]	RCT	83	24	MMV/MSV	40	45	63	67.3	
				MPP	43	99	63	68.2	
Tenholder et al. 2005 [50]	n-RCT	118		MMPP	69		56	66.8	29.3
				MPP	49		47	63.5	31.5
Thienpont et al. 2013 [51]	RCT	300	24	MMPP	150	100	67	68.0	30.4
				MPP	150	100	70	69.0	29.8
Tsuji et al. 2010 [53]	n-RCT	20	0.5	MMV	10	100	60	68.4	28.1
				MPP	10	100	80	69.8	28.9
Unnanuntana et al. 2012 [54]	n-RCT	64	60	MMPP	31				
				MPP	29				
Unwin et al. 2017 [55]	RCT	66	72	MMV/MSV	32		76	67.0	
				MPP	34		76	67.0	
Varela-Egocheaga et al. 2009 [56]	RCT	100	36	MSV	50		72	68.0	31.0
				MPP	50		74	70.6	30.6
Watanabe et al. 2009 [57]	n-RCT	48	48	MMV	25	84	80	71.0	28.1
				MPP	23	78	74	71.0	26.3
Wegrzyn et al. 2013 [58]	RCT	36	2	MSV	18	100	72	67.0	30.0
				MPP	18	100	72	64.0	31.0
Wülker et al. 2010 [60]	RCT	134	12	MSV	66	92	73	70.2	29.3
				MPP	68	88	70	29.3	
Zhu et al. 2015 [62]	n-RCT	67	109.2	MMPP	30		93	67.9	27.6
				MPP	37		84	65.3	27.7

*MMV* mini-midvastus, *MSV* mini-subvastus, *QS* quadriceps-sparing, *MMPP* mini-medial parapatellar, *MPP* medial parapatellar

**Table 3:** Demographic baseline of the two cohorts

Variable	MIS (*n* = 1697)	SIS (*n* = 1584)	*P*
Age (mean SD)	68.4 ± 2.8	67.7 ± 2.7	0.8
Female gender (%)	67%	67%	0.9
BMI (kg/m^2^)	29.4 ± 1.7	29.2 ± 1.8	0.9
OA patients (%)	97%	99%	0.9

### Outcomes of interest

**Fig. 3:**
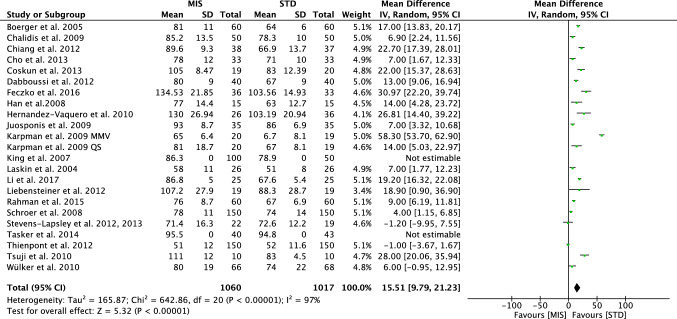
Forest plot of the comparison surgical duration

**Fig. 4:**
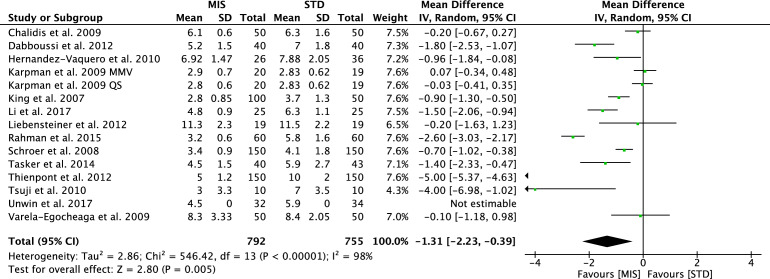
Forest plot of the comparison hospitalization length

The traditional SIS approach allows a shorter surgical duration (MD − 15.51; CI 9.79–21.23; *P* < 0.0001, Fig. 3). The MIS group was associated with a shorter hospitalization length (MD − 1.31; CI − 2.23 to − 0.39; *P* = 0.005, Fig. 4), a lower total estimated blood loss (MD − 76.88; CI − 183.35–29.58; *P* = 0.006) and quicker time of straight-leg raise (MD − 1.47; CI − 2.89 to − 0.05; *P* = 0.04).

**Fig. 5:**
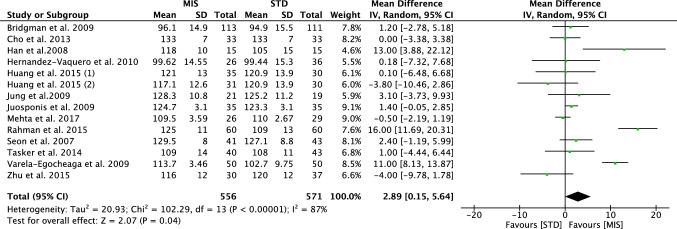
Forest plot of the comparison range of motion

At a mean follow-up of 21.31 ± 24.3 months, greater values of ROM were evidenced in the MIS group (MD 2.89; CI − 0.15–5.64; *P* = 0.04, Fig. 5), flexion (MD 5.92; CI 3.26–8.57; *P* < 0.0001), greater values of KSS (MD 1.09; CI 0.55–1.64; *P* < 0.0001) and KSFS (MD 3.07; CI 1.08–7.21; *P* = 0.01).

**Fig. 6:**
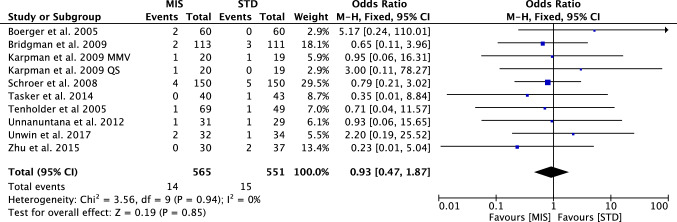
Forest plot of the comparison revision

The visual analogic scale, the rate of anterior knee pain and revisions (Fig. 6) were similar between the two cohorts. Table 4 shows the main results of the meta-analyses, while Table 5 displayed the complications.

**Table 4:** Main results of the comparisons

Outcome	Studies (*n*)	Knees (*n*)	MIS (mean ± SD)	SIS (mean ± SD)	Effect estimate, MD [95% CI]	*P*
Hospitalization length	15	1547	5.10 ± 2.4	6.71 ± 2.3	− 1.31 [− 2.23, − 0.39]	0.005
Surgical duration	23	2077	86.97 ± 20.2	75.69 ± 14.3	15.51 [9.79, 21.23]	< 0.0001
Total estimated blood loss	19	1499	601.23 ± 197.8	680.14 ± 300.6	− 76.88 [− 183.35, 29.58]	0.006
Straight-Leg Raise	6	630	2.12 ± 0.8	3.77 ± 1.9	− 1.47 [− 2.89, − 0.05]	0.04
ROM	14	1127	117.06 ± 10.8	113.71 ± 11.8	2.89 [0.15, 5.64]	0.04
Flexion	8	722	107.14 ± 11.6	104.79 ± 15.8	5.92 [3.26, 8.57]	< 0.0001
KSS	15	1411	89.69 ± 4.8	86.21 ± 5.5	1.09 [0.55, 1.64]	< 0.0001
KSFS	9	963	79.13 ± 3.5	75.41 ± 4.8	3.07 [− 1.08, 7.21]	0.01
VAS	8	738	2.12 ± 0.6	2.62 ± 1.2	− 9.25 [− 20.65, 2.14]	0.1

**Table 5:** Complications

Outcome	Studies (*n*)	Knees (*n*)	MIS (events)	SIS (events)	Effect estimate, OR [95% CI]	*P*
Anterior knee pain (OR)	2	169	21/258	14/249	3.54 [0.71, 17.55]	0.1
Revision surgery (OR)	10	1116	14/565	15/551	0.93 [0.47, 1.87]	0.9

## Discussion

The main finding of the present meta-analysis is that MIS approaches may offer clinical and functional benefits over the conventional MPP SIS approach for TKA. Peri-operatively, these include a significantly shorter length of hospitalization and time to straight-leg raise, along with a reduction in total estimated blood loss. Overall, patients undergoing MIS TKA achieve greater ROM, flexion and KSS and KSFS scores, and the surgical procedure is shorter. Concerning complications, the two approaches yielded similar results.

MIS TKA procedures have been introduced to minimize quadriceps disruption, resulting in better quadriceps strength [19, 41, 44]. Furthermore, the shorter incision and limited knee arthrotomy, and avoidance of patellar eversion and dislocation and hyperflexion of the tibiofemoral joint, produce less damage to the muscles, collateral ligament, and posterior capsule. All these features may result in faster recovery [12, 16]. However, given to the difficulty in execution, the longer learning curve and the need for special instruments, MIS TKA has not become very popular [2, 36]. The instrumentation for MIS TKA necessitates special retractors and jigs (e.g., the sided cutting tools). These instruments require adequate technical training. The revised sided cutting tool allow the coronal and sagittal bony cuts in one step, while, with the traditional instrumentation, two bone cuts are necessary. To assist the surgeon, the use of mobile windows can facilitate exposure of knee surfaces, and adequately trained assistants have also been recommended [26, 33, 52]. In addition to the new surgical instrumentation, new implants designed specifically for MIS TKA (e.g., the uncemented tibial plateau with smaller keel) have been introduced into the market, along with specific recoated stems and modular implants. These implants are designed specifically for those situations with reduced visibility of the surgical field. Several companies are introducing new uncemented implants to avoid improper cementation because of the small field of vision of MIS TKA [45]. Moreover, for MIS TKA, navigation systems, along with patient-specific instrumentation, recently gained popularity [21, 37, 61]. However, despite noticeable improvements, there are still controversial, and the reliability and feasibility, cost-effectiveness and clinical advantages of these new tools and new implants is uncertain. This has discouraged many surgeons from performing minimally-invasive TKAs, and the MPP remains the most common approach for TKA.

The reduction in hospitalization time for MIS patients offers great potentials for cost- savings. Notwithstanding, MIS approaches require a long learning curve for the whole surgical team [1, 31]. Reduced visibility of anatomical landmarks, the number of surgical steps, and the need for different equipment play a role in extending the duration of surgery. Once surgeons are more familiar with the less invasive procedures, operating times do decrease [11, 26], and eventually no difference in surgical time are found after surgeons received adequate training.

The endpoint total estimated blood loss was evaluated under a random effect method, given the high grade of heterogeneity. This can be explained by the different protocols of tourniquet, drainages and antifibrinolytic agents used in the various studies. Thus, even though this endpoint resulted statistically significant in favour of the MIS group, this result must be interpreted with caution. The time to straight-leg raise is used to assess functional recovery of the quadriceps muscle after a TKA; the statistically significant reduction in time detected among the MIS group is noteworthy. The faster restoration of function of the extensor muscle mechanism may arise from to the limited knee arthrotomy and smaller incision in MIS TKA, together with the avoidance of patellar eversion. Reduced damage to soft tissues may also explain the statistically significant improvement of the analysed scores. The visual analogic scale for pain, even if not statistically significant, was remarkably lower in the MIS group. Similar consideration can be inferred also to the KSS and KSFS, which resulted statistically significant better outcomes in favour of the MIS group. A statistically significant improvement of joint motion was observed (ROM and flexion). Some studies found that MIS TKA resulted in an improvement of ROM and flexion in the early post-operative period, which disappeared after one week and three months [3, 32]. During TKA performed by SIS approach, the quadriceps tendons and muscles are incised and re-sutured, resulting in scar tissues and fibrosis, which can explain the reduced joint motion. However, evidences are lacking, and future studies should investigate and compare the trend of favourable joint motion in MIS over the time. This study encourages orthopaedic surgeons to consider MIS TKAs approaches, notwithstanding the difficulties that arise from the longer learning curve. In light of the present results, further studies should investigate the best approach for MIS TKA and establish with greater stringency what the correct indications for MIS TKA are.

## Limitations

Given of the high overall heterogeneity, all comparisons were analysed under a random effect method. Moreover, the funnel plot detected a moderate risk of publication bias. Articles were compared regardless to the type of pre- and peri-operative protocols. The patient anatomical characteristics, time of tourniquet, use of antifibrinolytic agents and antibiotics administration, type of technique, type of implants, use of drainages and post-operative rehabilitation and antithrombotic protocols were source of major differences which could not be adjusted statistically. Limited follow-up times represent another important limitation of this study, resulting in unreliable data concerning long-term complications and implant survivorship. A lack of distinction between MIS approaches (QS, MMV, MSV, MMPP), a reflection of the available data, represent an important limitation of this study. This was necessary to improve the amount of pooling data for inclusion. Further studies are required to investigate the pros and cons of the various approaches separately when enough suitable studies will have been published. Another important limitation of this study is the number of analysed endpoints, which was limited by insufficient data in the literature which would allow for analysis of further endpoints. Computer-assisted TKA were not considered, and this may represent another limitation. Moreover, limitations in reported durations of follow-up prevents a more robust analysis of long-term benefits and risks. Given these limitations, results from the present study must be interpret with caution.

Strong points of the present work, on the other hand, are represented by the widespread nature of the literature search, along with the strict eligibility criteria, its methodological quality assessment, and good baseline comparability. This is crucial to provide more reliable and homogeneous results, leading to greater strength of scientific evidence.

## Conclusion

MIS approaches may offer clinical and functional benefits over conventional SISI MPP approach for TKA. Peri-operatively, MIS patients experience lower total estimated blood loss and a reduction in hospitalization time. Post-operatively, MIS patients demonstrate improved joint function and other outcome scores during follow-up. Minimally invasive approaches for TKA involve a technically more complicated execution which requires a long learning curve for the whole surgical team. These results must be interpreted within the limitations of the present study.
